# 

**Published:** 1892-07-09

**Authors:** 


CONTENTS.

The Ideals of Science . . . . . . . . . . 241

Passing Topics.—The Lords and the Nurses . . . . . 242

The Doctor's Armchair.—Some "Bloated Aristocrats" of Physic . . . . . . . . . . 243

Bill Sykes's Son . . . . . . . . . . 244

Editor's Letter Box.—Is Vivisection Immoral? . . . . 244

Medical History from the Earliest Times. By E. T. WITHINGTON, M.A., M.B.Oxon. XVI.—The Medical Profession in Anc ent Greece . . . . . . . . . . 245

Everybody's Page . . . . . . . . . . 246

Annotations.—The Masculine Fringe—Mr. James Payn's Physicians—A Paradise for Consumptives . . . . . 247

Notes and News . . . . . . . . . . 248

Around the Hospitals . . . . . . . . . . 249

The Student of Medicine.—Appointments—Vacancies—Notes from the Schools—University Inte'ligence

Scraps and Gleanings . . . . . . . . . . 249

The Practitioners' Mirror and Retrospect—

MEDICINE.—The Death-rate of Pneumonia . . . . . 250

SURGERY.—On the Treatment of Cystic Goitre . . . . 250

OPHTHALMOLOGY.—Hypermetropic Headaches . . . . 251

PRACTITIONERS' BOOKSHELF.—Materia Medica . . . . 252

NEW DRUGS, APPLIANCES, AND THINGS MEDICAL.—Mellin's Food 252

The Institutional Workshop—

HOSPITAL ADMINISTRATION. II.—Admission of Patients . 253

WITHIN THE HOSPITALS. IV.—The Royal Hants County Hospital, Winchester . . . . . . . . . . 254

PRACTICAL POINTS . . . . . . . . . . 254

HOSPITAL CONSTRUCTION.—"Barraque" Wards . . . . 255

HOSPITAL OPINION—Special Hospitals . . . . . 255

INSTITUTION LITERATURE.—Asylums of the World. II.—Treatment . . . . . . . . . . 255

EXTRA SUPPLEMENT.—THE NURSING MIRROR.

En Passant.—Holidays—Cheltenham General Hospital—The Holborn Guardians—Short Items—Prize-giving at School of Medicine for Women—The Q.V.J.I.N. in Scotland—Nurses for India—Invalid Children's Aid Association—Stalybridge Sick Nursing and Mothers' Help Society . . . . ci

Ventilation, Disinfection, and Diet. By P. CALDWELL SMITH. XIII.—Diet and Dietaries . . . . . . cii

Select Committee on Metropolitan Hospitals . . . ciii

How to Procure Nurses for India . . . . . . civ

Asylum Notes . . . . . . . . . . cv

Everybody's Opinion.—Nurse's Holidays and King's College Hospital—Hamilton Association for Nurses—Jersey . . cv

Queen Victoria's Jubilee Institute for Nurses . . . cv

Wants and Workers . . . . . . . . . . cv

Four Months in a Hospital Ward (*concluded*) . . . . cvi

Notes and Queries . . . . . . . . . . cvi

